# Intradural extraarachnoid sutureless technique combined with laminoplasty for indirect repair of ventral dural defects in spontaneous intracranial hypotension: technical note and case series

**DOI:** 10.1007/s00701-021-04868-2

**Published:** 2021-05-08

**Authors:** M. Kamenova, S. Schaeren, M-G. Wasner

**Affiliations:** Department of Spine Surgery, University Hospital of Basel, Spitalstrasse 21, 4031 Basel, Switzerland

**Keywords:** CSF leak, Microspurs, Posterior approach, Spinal dural tears, Spontaneous intracranial hypotension

## Abstract

**Background:**

There is a significant variance in surgical treatment strategies of ventral cerebrospinal fluid (CSF) leaks causing spontaneous intracranial hypotension (SIH). Posterior approaches might represent a preferable alternative to the more invasive anterior and lateral routes, as long as the spinal cord is not exposed to harmful manipulation. The aim of this technical note is to report and illustrate a new surgical technique using an intradural extraarachnoid sutureless technique via laminoplasty for indirect repair of ventral CSF leaks causing intractable SIH symptoms.

**Methods:**

The surgical technique is described in a step by step fashion. Between May 2018 and May 2020, five patients with ventral spinal CSF leaks were operated on, utilizing this technique. All dural defects were located at the level of the thoracic spine. A retrospective review on demographic and radiological findings, symptoms, outcome, and follow-up was performed.

**Results:**

The intra- and postoperative course was uneventful in all patients with no surgery-related complications. Three patients recovered completely at discharge, while neurological symptoms significantly improved in two patients. A postoperative MRI of the spine was obtained for all patients, demonstrating regressive signs of CSF leak.

**Conclusion:**

Based on the presented case series, this intradural extraarachnoid sutureless technique combined with laminoplasty seems to be a safe and effective option for indirect repair of ventral dural defects in SIH. In our opinion, it represents a valid alternative to traditional more aggressive approaches.

## Introduction

Spontaneous intracranial hypotension is an uncommon but well-recognized cause of headache with an estimated prevalence of 1/50,000 individuals [14]. Women are more commonly affected than men and symptoms mostly occur in the fourth and fifth decade of life [3, 6, 14, 17]. Patients typically present with orthostatic headache, caused by a spinal cerebrospinal fluid (CSF) leak [2, 10, 15]. Most cases resolve spontaneously or respond well to epidural blood patches (EBP) [16]; however, in patients refractory to conservative treatment and a known location of the CSF leak, surgery represents a safe and effective treatment [1, 14, 16, 18].

Dural tears are mostly located anteriorly [1, 2] which represents a significant surgical challenge. To date, given the few studies available, there is no consensus regarding the optimal approach for the repair of ventral CSF leaks. The traditional access to the ventral dura includes extensive anterior and lateral approaches aiming to reduce potential risk to the spinal cord as they provide a direct route to the CSF leaks [1, 4]. On the other hand, posterior approaches have been proposed [1, 5, 11–13]. Still, there is a significant variety in the surgical nuances and grade of invasiveness.

According to our experience, posterior approaches might represent a more elegant alternative for addressing ventral CSF leaks, although they challenge conventional practice favoring transpedicular approaches and costotransversectomy, as well as anterior or lateral approaches.

In order to minimize manipulation of the spinal cord, we propose an intradural extraarachnoid sutureless technique for indirect repair of ventral CSF leaks. With this work, we aim to illustrate a novel surgical technique which in our case series of five patients showed promising results without any surgery-related complications.

## Methods

### Case series

Consecutive patients with ventral CSF leaks who were treated surgically for SIH between May 2018 and May 2020 at the Department of Spine Surgery at the University Hospital of Basel were identified retrospectively. The medical records of the patients were reviewed to extract demographic data, symptoms, radiological findings, operative details, and postoperative follow-up. A general informed consent was signed by all patients. The study protocol was approved by the local ethics committee (EKNZ, Basel, Switzerland).

### Patient characteristics and diagnostic findings

Five cases were diagnosed with SIH according to the criteria of the International Classification of Headache Disorders, Third Edition (ICHD, Headache Classification) [8] and treated surgically during the study period. All operated patients suffered from intractable SIH symptoms after failure of conservative treatment. Four patients (80%) were women, and the mean age at presentation was 50.2 ± 8.8 years. All but one patient received a diagnostic work-up including cranial MRI, MRI of the spinal axis, myelography, and postmyelography CT imaging of the spine as well as measurement of the lumbar opening pressure. In one patient, a myelography was not necessary. In all patients, a calcification of the PLL with a ventral epidural CSF leakage was found at the level of the intervertebral disk space at the thoracic spine. These calcifications were preoperatively identified on the CT scan imaging and defined our target level during surgery. Demographic data, preoperative symptoms, levels of pathology, and rates of application of EBP are presented in Table 1.

**Table 1:** Patient characteristics and diagnostic findings

Patients	Age (y)	Sex	Level of dural defect	Pathology	EBP	EBP (*n*)	Preoperative symptoms
1	43	F	T2/3	Calcification of PLL	Yes	1	1, 5, 6
2	61	M	T9/10	Calcification of PLL	Yes	1	1, 2, 3
3	61	F	T8/9	Calcification of PLL	No	0	1, 4, 6, 7, 8
4	44	F	T11/12	Calcification of PLL	Yes	2	1
5	42	F	T11/12	Calcification of PLL	Yes	4	1

*y* years, *PLL* posterior longitudinal ligament, *n* number, *EBP* epidural blood patch

1, orthostatic headache; 2, cervical myelopathy; 3, motor deficit; 4, tinnitus; 5, gait disturbance; 6, vomitus; 7, vertigo; 8, hypacusis

### Microsurgical procedure

**Fig. 1:**
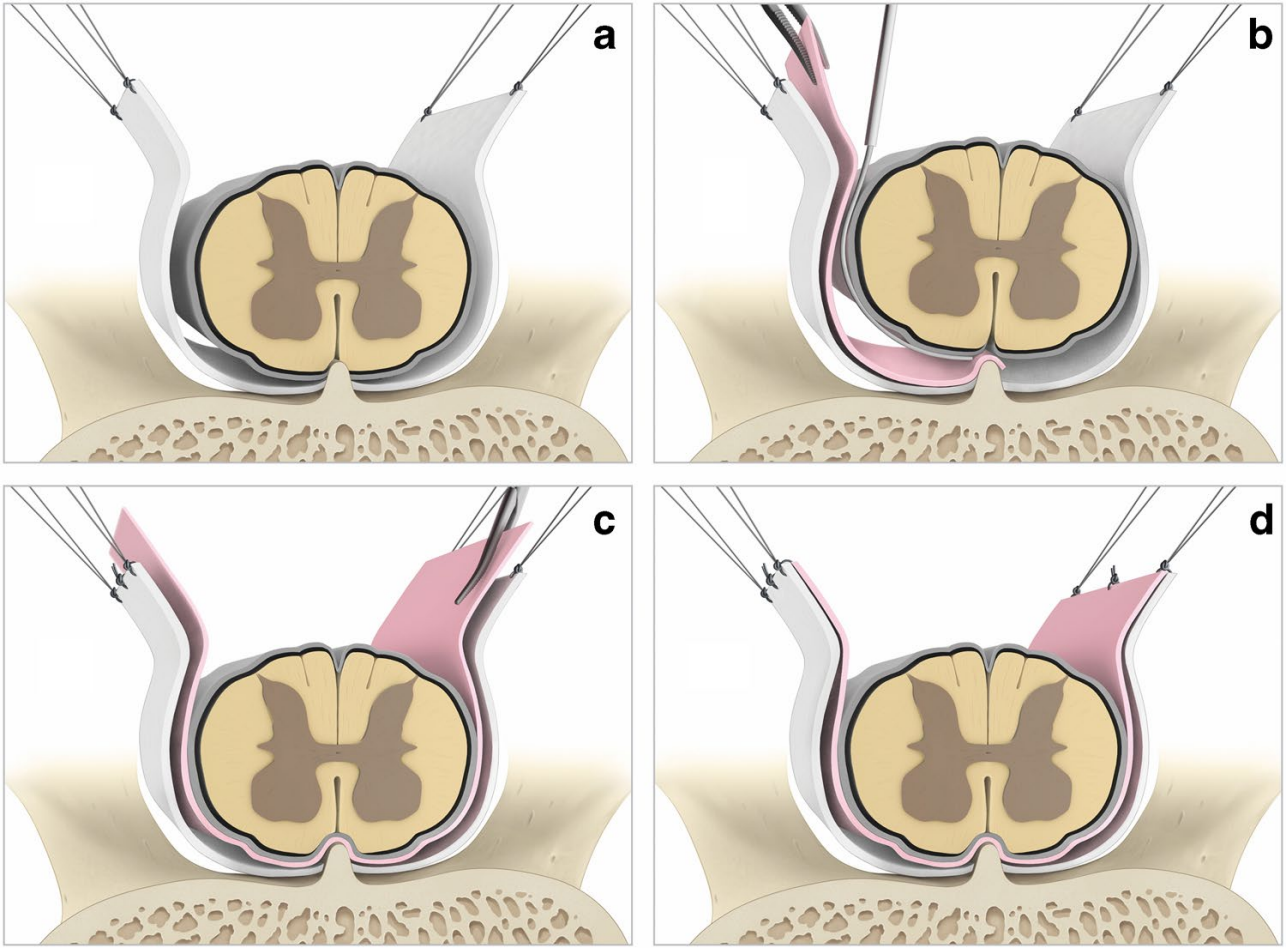
Main steps of the intradural extraarachnoid technique for indirect repair of ventral CSF leaks. **a** A dorsal midline durotomy without injuring the arachnoidea is performed. The edges of the dura are tacked up with stitches on each side. **b** Gentle elevation of the spinal cord to open the subdural space and introduce the GORE-TEX® Cardiovascular Patch between the dura and the arachnoid layer. **c** The graft must be large enough to overlap with the normal dura around the defect. **d** Both ends of the graft are cut accordingly; then, two tacking sutures were placed on each side, in order to fix the graft to the dural edges

**Fig. 2:**
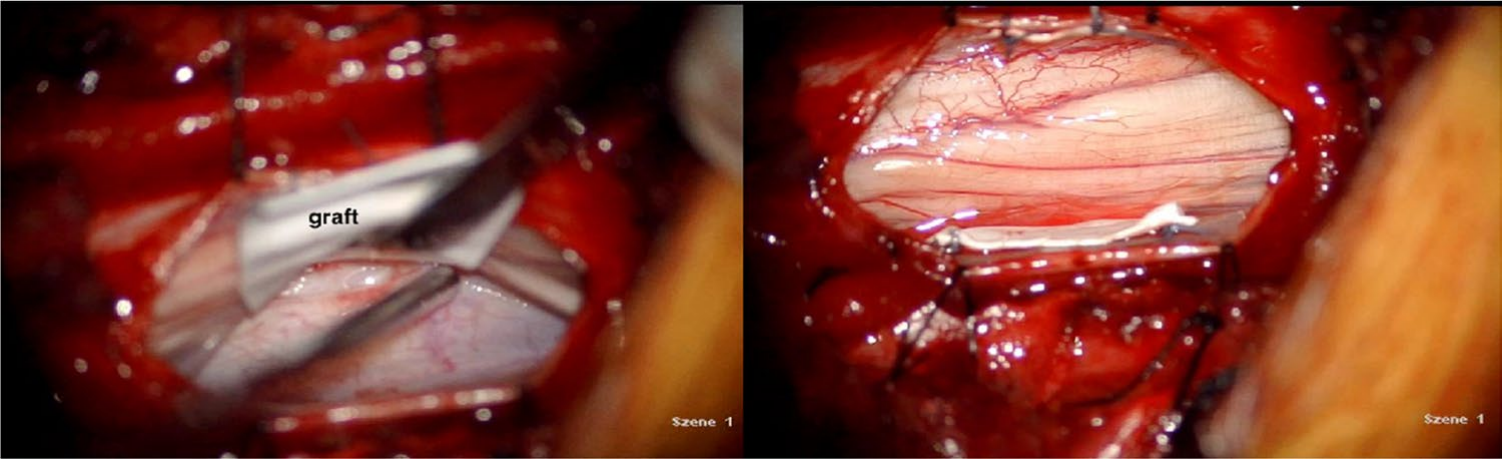
Intraoperative image showing the graft, which is gently introduced between the dural defect and the arachnoidea, and intraoperative image showing the graft which is already cut accordingly and fixed with tacking sutures on each side

Surgery was performed in the prone position under general anesthesia, after identifying the index level by fluoroscopy. In all patients, intraoperative neurophysiological monitoring (IOM) of motor evoked potentials (MEP), somatosensory evoked potentials (SSEP), and free running electromyography (EMG) was installed with the established parameters and settings. After a midline incision, a single laminotomy was carried out using an oscillating saw and the yellow ligament was removed. The cranial or caudal lamina was undercut if necessary. Surgery was continued under microscopic view. A dorsal midline durotomy was performed, without injuring the arachnoid layer. The edges of the dura were then tacked up with stitches on each side (Fig. 1a). The arachnoid layer around the spinal cord was slightly elevated using microsurgical tweezers, so that the border between arachnoidea and dura was exposed. A plane of dissection between the arachnoid layer and the dura was then performed using micro-raspartories and micro- and disk dissectors from the lateral to the ventral aspect of the subdural space. The dentate ligament was cut. After gentle exploration using micro-dissectors, the ventral dural defect was identified in all patients at the preoperatively expected location, where an associated calcification of the PLL was found. These calcifications were not removed in all but one cases (the first one in our case series). A dry GORE-TEX® Cardiovascular Patch (Gore Medical, Flagstaff, AZ, USA) (thickness 0.4 mm, width × length 2 cm × 9 cm) was cut accordingly with one ending narrower than the other, making sure that it is large enough to overlap with normal dura both around the defect in the cranio-caudal and in the lateral side-to-side direction. The spinal cord and the arachoideal layer were then gently elevated to open the subdural extraarachnoid space. The dry graft was then introduced with the narrow ending ahead (allowing easier manipulation) between the dura and the arachnoid layer and slid around, from the ipsi- to the contralateral side using microsurgical tweezers and microsurgical dissectors (Fig. 1b; Fig. 2). Both ends of the graft were cut accordingly; then, two tacking sutures were placed on each side, in order to fix the graft to the dural edges (Fig. 1c, d; Fig. 2). After intradural repair, the dura was closed in a watertight fashion, using a TiCron™ polyester non-absorbable suture. The lamina was then reattached and secured using titanium miniplates and screws, after placing of two dural tenting sutures in order to prevent potential epidural hematomas. No drains were used. Finally, the wound was closed respecting the anatomic layers.

## Results

### Clinical outcome and postoperative follow-up

None of the patients experienced postoperative surgical complications. At discharge, three patients recovered completely from SIH, while in two patients symptoms improved. One of these two patients suffered not only from orthostatic headache but also from cervical myelopathy symptoms and coordination disorders, so some residual motor deficits persisted postoperatively. The other patient with history of migraine had some non-orthostatic headaches at discharge. All patients had a MRI imaging of the spine at follow-up, while one patient additionally received a CT scan. No signs for residual CSF leaks were seen. At follow-up, three patients were still asymptomatic, while the two other patients had slight residual, non-disabling symptoms. Operation and hospitalization time as well as outcome data at discharge and follow-up are demonstrated in Table 2.

**Table 2:** Patient outcome and postoperative follow-up

Patients	OR time (min)	Surgical complications (*n*)	Hospitalization time (d)	Symptoms at discharge (0 = none, 1 = better, 2 = worse, 3 = unchanged)	mRS at discharge	Last clinical FU (d postop.)	Last MRI spine at FU (d postop.)	MRI result (0 = no signs for CSF leak, 1 = residual CSF leak)	Symptoms at FU (0 = no symptoms, 1 = slight residual symptoms)
1	137	0	6	0	0	88	88	0	0
2	133	0	6	1	1	105	105	0	1
3	150	0	13	0	0	928	928*	0	0
4	150	0	5	0	0	176	175	0	0
5	105	0	8	1	0	51	51	0	1

*OR time* operation time, *n* number, *d* days, *mRS* modified Rankin Scale, *FU* follow-up

^*^Additional CT scan of the thoracic spine

## Discussion

Besides the minimal invasiveness requiring a single laminoplasty, the main advantage of our technique is that neither rotation of the spinal cord nor spur removal or risky primary repair of the dura is necessary. In the first patient of our series, we removed the calcification of the PLL, which lead to a transient deterioration of MEPs. Even though the patient had no neurological deficits postoperatively, we decided to abandon this step since then. The calcifications of the PLL in our series were neither sharp nor “knife-like” or bony, and the dural defects were rather oval-shaped and not slit-like, so actually we think that they develop secondarily as a soft tissue reaction to the chronic pulsating CSF pressure through the dural defect, while the arachnoid layer was still intact. As the outcome of the next patients was still excellent, and improvement of the symptoms or complete recovery was achieved, we do not think that exposing the spinal cord to further risk by removing these calcifications is justified. The effectiveness of the technique was also confirmed by follow-up imaging showing no signs of spinal CSF leak. The graft that is slid around the dura seems to be sufficient, given the clinical and radiological results mentioned above. Even if microspurs or sharper ossifications are found, we believe that a Gore-Tex patch represents a stable barrier for covering the leakage. Moreover, technically demanding primary dural repair with the risk of suture insufficiency and spinal cord manipulation can be avoided. It is also important to emphasize that putting the graft into the extraarachnoid space is a crucial step of our technique. In this way, both potential scarring and hazard to the anterior spinal artery can be prevented and a safe barrier between the graft and the spinal cord is created.

Beck et al. recently proposed a posterior approach with spinal cord release maneuver, including cutting the dentate ligament and gentle rotation and elevation of the spinal cord [1]. Surgical complication rate was 28% while 14% of the patients with ventral leaks experienced transient neurologic deficits. The authors themselves attributed these to the manipulation of the spinal cord. When microspurs penetrating the dura were found, these were removed and the dura was readapted. However, two cases of suture insufficiencies with recurrent symptomatic SIH led to revision surgery. Although at first sight this seems to be an elegant technique, obviously even gentle rotation of the spinal cord might be crucial, as 14% of the patients had neurological symptoms postoperatively. Even if these were transient, we believe that every unnecessary risk to the spinal cord should be avoided.

Similar techniques using a graft (collagen matrix graft “DuraGen Plus” and bovine pericardium) with and without tacking sutures have been proposed for the treatment of transdural herniations of thoracic spinal cord [7, 9] These approaches were more invasive as resection of the pedicle and the transverse process were necessary. The grafts were placed “intradurally” and the authors do not detail if the arachnoid layer was respected. Moreover, these techniques have not been proposed for ventral dural leaks due to SIH by now.

In summary, the optimal surgical approach to the ventral dura should be minimally aggressive, effective, and safe. In our experience, the posterior intradural extraarachnoid sutureless technique combines all these features. Clearly, a larger cohort will be needed to confirm our results.

### Limitations

As in any case series, definitive conclusions regarding the true efficacy of this technique cannot be made, especially with our small cohort of five patients. However, considering the rarity of the disease, it is difficult to obtain large cohorts or perform randomized trials.

Follow-up was mostly relatively short, without standardized outcome parameters, and the only follow-up imaging performed was a MRI of the spine (in all but one patient who also received a CT scan). However, as in all patients symptoms completely resolved or improved, we do not think that further imaging or long-term follow-up was necessary. The most important measurements on complications, neurologic deficits, improvement of symptoms, and follow-up imaging were well-documented and show promising results.

Lastly, as mentioned in the “Discussion” section, similar (but more invasive) approaches have been described for other pathologies. However, this is the first technical note on using an intradural and extraarachnoid technique for indirect repair of ventral dural defects.

## Conclusion

Based on the presented case series, this intradural extraarachnoid sutureless technique combined with laminoplasty seems to be a safe and effective option for indirect repair of ventral dural defects in SIH. In our opinion, it represents a valid alternative to traditional more aggressive approaches.
